# Supplementary material for: Natural History of Aerosol Induced Lassa Fever in Non-Human Primates
Source: Viruses. 2020 May 29;12(6):593. doi: 10.3390/v12060593 (PMC7354473; doi:10.3390/v12060593)
Supplement: Supplementary file 1 [file viruses-12-00593-s001.pdf]

Table S1. Clinical Assessment Score of Non-Human Primates during the Natural History Study.

	GROUPS	DPE 0	DPE 1	DPE 2	DPE 3	DPE 4	DPE 5	DPE 6	DPE 7	DPE 8	DPE 9	DPE 10	DPE 11	DPE 12	DPE 13	DPE 14	DPE 15	DPE 16	DPE 17	DPE 18	DPE 19	DPE 20	DPE 21
MEAN CLINICAL SCORES	ALL ANIMALS	0	0	0	0	0	0.13	0.25	0.31	0.42	0.60	0.93	1.00	1.21	1.63	0.83	1.17	0.58	0.58	0.67	0.67	0.67	0.42
STAGE OF DISEASE	ALL ANIMALS	SUBCLINICAL	SUBCLINICAL	SUBCLINICAL	SUBCLINICAL	CLINICAL	CLINICAL	CLINICAL	CLINICAL	DECOMP	DECOMP	DECOMP	DECOMP	DECOMP	DECOMP	DECOMP	DECOMP	DECOMP	DECOMP	DECOMP	DECOMP	DECOMP	DECOMP
Responsiveness	Survivor	0	0	0	0	0	0	0	0	1	2	2	2	2	2	1	2	1	1	1	1	1	0
/Behavior	Non-survivors	0±0	0±0	0±0	0±0	0±0	0.33±0.58	0.33±0.59	0.33±0.60	0.33±0.61	1±1	2±1.73	1±0	1±0	3±0	N/A	N/A	N/A	N/A	N/A	N/A	N/A	N/A
Rash	Survivor	0	0	0	0	0	0	0	0	0	0	0	0	0	0	0	0	0	0	0	0	0	0
	Non-survivors	0±0	0±0	0±0	0±0	0±0	0±0	0±0	0±0	0±0	0±0	0±0	0±0	0±0	1±0	N/A	N/A	N/A	N/A	N/A	N/A	N/A	N/A
Bleeding	Survivor	0	0	0	0	0	0	3	0	3	3	3	3	3	3	0	3	0	0	0	0	0	0
	Non-survivors	0±0	0±0	0±0	0±0	0±0	0±0	0±0	0±0	0±0	0±0	0±0	0±0	3±0	3±0	N/A	N/A	N/A	N/A	N/A	N/A	N/A	N/A
GI Symptoms	Survivor	0	0	0	0	0	0	1	1	1	3	N/A	2	3	3	3	3	3	3	3	3	3	3
	Non-survivors	0±0	0±0	0±0	0±0	0±0	0±0	0.33±0.58	0.67±0.58	0.67±0.58	0.67±0.58	N/A	2±0	2±0	2±0	N/A	N/A	N/A	N/A	N/A	N/A	N/A	N/A
Food Consumption	Survivor	0	0	0	0	0	0	0	2	2	2	2	3	3	3	3	2	2	1	1	1	1	1
	Non-survivors	0	0	0	0	0	1±1	1±1	2±0	2±0	2±0	3±1	3±0	3±0	3±0	N/A	N/A	N/A	N/A	N/A	N/A	N/A	N/A
Urine Output	Survivor	0	0	0	0	0	0	0	0	0	1	0	1	1	1	1	1	0	0	0	0	0	0
	Non-survivors	0±0	0±0	0±0	0±0	0±0	0.67±0.58	1±0	1±0	1±0	1±0	1±0	1±0	1±0	1±0	N/A	N/A	N/A	N/A	N/A	N/A	N/A	N/A
Posture	Survivor	0	0	0	0	0	0	0	0	0	2	2	3	3	3	1	2	1	1	1	1	1	0
and Appearance	Non-survivors	0±0	0±0	0±0	0±0	0±0	0±0	0±0	0±0	0.33±0.58	0.33±0.58	2.33±2.08	2±0	2±0	4±0	N/A	N/A	N/A	N/A	N/A	N/A	N/A	N/A
Edema	Survivor	0	0	0	0	0	0	0	0	0	0	0	0	0	0	0	0	0	0	0	0	0	0
	Non-survivors	0±0	0±0	0±0	0±0	0±0	0±0	0±0	0±0	0±0	0±0	0±0	0±0	0±0	3±0	N/A	N/A	N/A	N/A	N/A	N/A	N/A	N/A
Respiration	Survivor	0	0	0	0	0	0	0	0	0	0	0	0	0	0	0	0	0	0	0	0	0	0
	Non-survivors	0±0	0±0	0±0	0±0	0±0	0±0	0±0	0±0	0±0	0±0	0±0	0±0	0±0	0±0	N/A	N/A	N/A	N/A	N/A	N/A	N/A	N/A
Exudate	Survivor	0	0	0	0	0	0	0	0	0	0	0	0	0	0	0	0	0	0	0	0	0	0
	Non-survivors	0±0	0±0	0±0	0±0	0±0	0±0	0±0	0±0	0±0	0±0	0±0	0±0	0±0	1±0	N/A	N/A	N/A	N/A	N/A	N/A	N/A	N/A
Neurological Function	Survivor	0	0	0	0	0	0	0	0	0	1	1	1	1	1	1	1	0	0	1	1	1	1
	Non-survivors	0±0	0±0	0±0	0±0	0±0	0±0	0±0	0±0	0±0	0±0	1.67±1.53	0±0	0±0	1±0	N/A	N/A	N/A	N/A	N/A	N/A	N/A	N/A
Vocalization	Survivor	0	0	0	0	0	0	0	0	0	0	0	0	0	0	0	0	0	1	1	1	1	0
	Non-survivors	0±0	0±0	0±0	0±0	0±0	0±0	0±0	0±0	0±0	0±0	0.33±0.58	0±0	1±0	1±0	N/A	N/A	N/A	N/A	N/A	N/A	N/A	N/A

	GROUPS	DPE 22	DPE 23	DPE 24	DPE 25	DPE 26	DPE 27	DPE 28	DPE 29	DPE 30	DPE 31	DPE 32	DPE 33	DPE 34	DPE 35	DPE 36	DPE 37	DPE 38	DPE 39	DPE 40	DPE 41
MEAN CLINICAL SCORES	ALL ANIMALS	0.42	0.42	0	0	0	0	0	0	0	0	0	0	0	0	0	0	0	0	0	0
STAGE OF DISEASE	ALL ANIMALS	DECOMP	DECOMP	DECOMP	DECOMP	DECOMP	DECOMP	DECOMP	DECOMP	RECOVERY	RECOVERY	RECOVERY	RECOVERY	RECOVERY	RECOVERY	RECOVERY	RECOVERY	RECOVERY	RECOVERY	RECOVERY	RECOVERY
Responsiveness	Survivor	0	0	0	0	0	0	0	0	0	0	0	0	0	0	0	0	0	0	0	0
/Behavior	Non-survivors	N/A	N/A	N/A	N/A	N/A	N/A	N/A	N/A	N/A	N/A	N/A	N/A	N/A	N/A	N/A	N/A	N/A	N/A	N/A	N/A
Rash	Survivor	0	0	0	0	0	0	0	0	0	0	0	0	0	0	0	0	0	0	0	0
	Non-survivors	N/A	N/A	N/A	N/A	N/A	N/A	N/A	N/A	N/A	N/A	N/A	N/A	N/A	N/A	N/A	N/A	N/A	N/A	N/A	N/A
Bleeding	Survivor	0	0	0	0	0	0	0	0	0	0	0	0	0	0	0	0	0	0	0	0
	Non-survivors	N/A	N/A	N/A	N/A	N/A	N/A	N/A	N/A	N/A	N/A	N/A	N/A	N/A	N/A	N/A	N/A	N/A	N/A	N/A	N/A
GI Symptoms	Survivor	3	3	0	0	0	0	0	0	0	0	0	0	0	0	0	0	0	0	0	0
	Non-survivors	N/A	N/A	N/A	N/A	N/A	N/A	N/A	N/A	N/A	N/A	N/A	N/A	N/A	N/A	N/A	N/A	N/A	N/A	N/A	N/A
Food Consumption	Survivor	1	1	0	0	0	0	0	0	0	0	0	0	0	0	0	0	0	0	0	0
	Non-survivors	N/A	N/A	N/A	N/A	N/A	N/A	N/A	N/A	N/A	N/A	N/A	N/A	N/A	N/A	N/A	N/A	N/A	N/A	N/A	N/A
Urine Output	Survivor	0	0	0	0	0	0	0	0	0	0	0	0	0	0	0	0	0	0	0	0
	Non-survivors	N/A	N/A	N/A	N/A	N/A	N/A	N/A	N/A	N/A	N/A	N/A	N/A	N/A	N/A	N/A	N/A	N/A	N/A	N/A	N/A
Posture	Survivor	0	0	0	0	0	0	0	0	0	0	0	0	0	0	0	0	0	0	0	0
and Appearance	Non-survivors	N/A	N/A	N/A	N/A	N/A	N/A	N/A	N/A	N/A	N/A	N/A	N/A	N/A	N/A	N/A	N/A	N/A	N/A	N/A	N/A
Edema	Survivor	0	0	0	0	0	0	0	0	0	0	0	0	0	0	0	0	0	0	0	0
	Non-survivors	N/A	N/A	N/A	N/A	N/A	N/A	N/A	N/A	N/A	N/A	N/A	N/A	N/A	N/A	N/A	N/A	N/A	N/A	N/A	N/A
Respiration	Survivor	0	0	0	0	0	0	0	0	0	0	0	0	0	0	0	0	0	0	0	0
	Non-survivors	N/A	N/A	N/A	N/A	N/A	N/A	N/A	N/A	N/A	N/A	N/A	N/A	N/A	N/A	N/A	N/A	N/A	N/A	N/A	N/A
Exudate	Survivor	0	0	0	0	0	0	0	0	0	0	0	0	0	0	0	0	0	0	0	0
	Non-survivors	N/A	N/A	N/A	N/A	N/A	N/A	N/A	N/A	N/A	N/A	N/A	N/A	N/A	N/A	N/A	N/A	N/A	N/A	N/A	N/A
Neurological Function	Survivor	1	1	0	0	0	0	0	0	0	0	0	0	0	0	0	0	0	0	0	0
	Non-survivors	N/A	N/A	N/A	N/A	N/A	N/A	N/A	N/A	N/A	N/A	N/A	N/A	N/A	N/A	N/A	N/A	N/A	N/A	N/A	N/A
Vocalization	Survivor	0	0	0	0	0	0	0	0	0	0	0	0	0	0	0	0	0	0	0	0
	Non-survivors	N/A	N/A	N/A	N/A	N/A	N/A	N/A	N/A	N/A	N/A	N/A	N/A	N/A	N/A	N/A	N/A	N/A	N/A	N/A	N/A

Table S1. Clinical Assessment Score of Non-Human Primates during the Natural History Study. Score codes during clinical assessment are normal (0), very mild (1), mild (2), moderate (3), and severe (4) for responsiveness/behavior, rash, bleeding, gastrointestinal (GI) symptoms, posture/appearance, and respiration. Score codes during the assessment are normal (0), mild (1), moderate (2), and severe (3) for food consumption, urine output, and edema. Vocalization assessment includes normal (0) and in distress (1). Exudate assessment includes normal (0) and present (1). Non-survivors (N=3, mean ± SD) group was compared to ●Survivor (N=1). Indicators for stages of disease include mean clinical score of all animals and additional analysis. Subclinical stage of disease progression (DPE 0-3) is marked by mean clinical score of zero from the day of challenge until presentation of fever and virus. Identification of the clinical stage of infection (DPE 4-7) involved detection of fever and virus. The decompensated stage (DPE 8-29) was identified with mean clinical score ≥ 0.4, peak viremia, alkalosis with blood pH > 7.5, and elevated liver and kidney functions marker levels. Recovery stage (Post-DPE 30) was establishing based on mean clinical score of zero for eleven days, viremia below 100 PFU/mL, blood pH <7.5, and liver and kidney functions markers near baseline level.

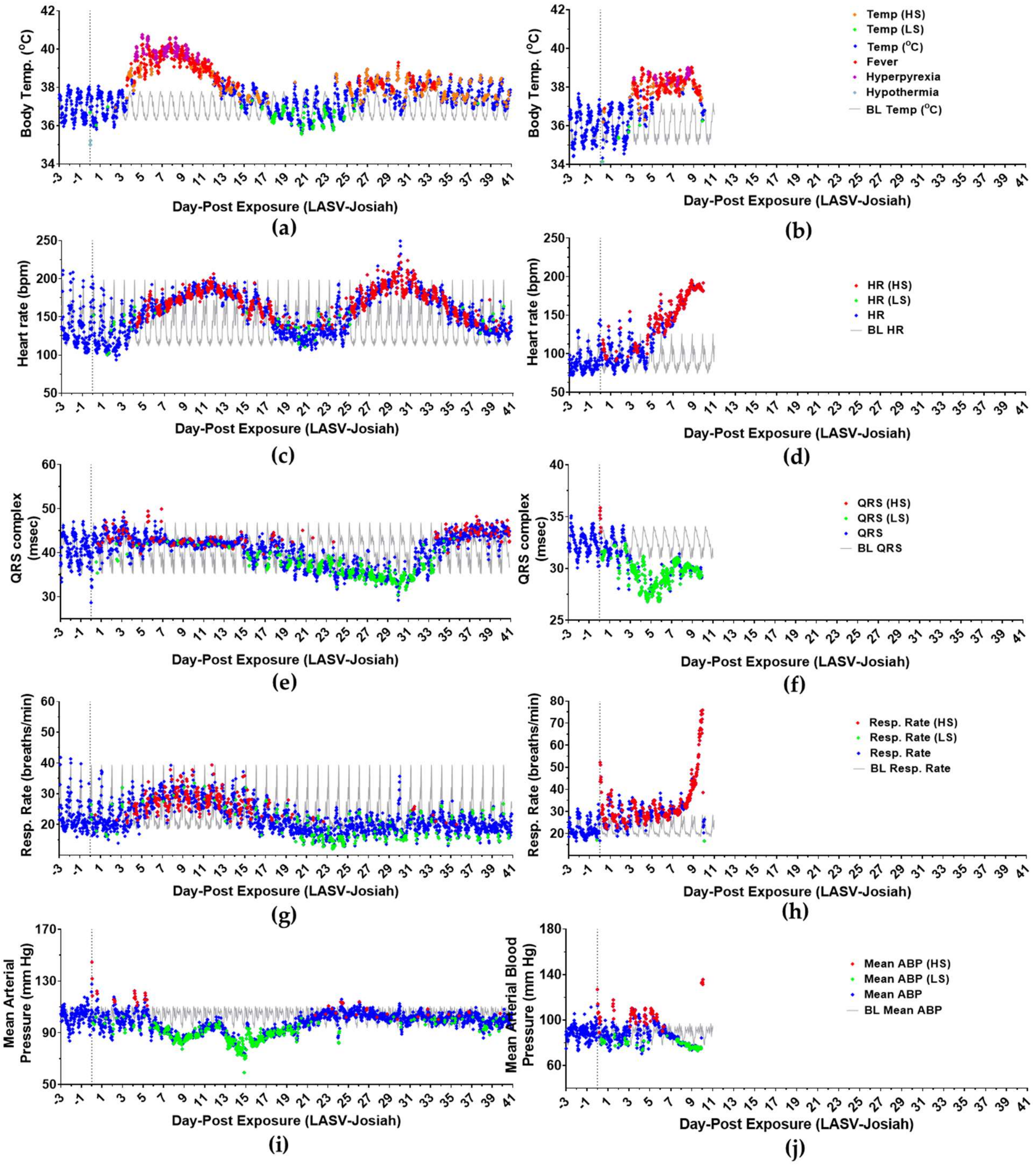

Figure S1. Telemetric assessment of aerosol Lassa virus infected cynomolgus macaques. (a) Temperature of survivor, (b) temperature of non-survivor, (c) heart rate of survivor, (d) heart rate of non-survivor, (e) QRS complex of survivor, (f) QRS complex of non-survivor, (g) respiratory rate of survivor, (h) respiratory rate of non-survivor, (i) mean arterial blood pressure of survivor, and (j) mean arterial blood pressure of non-survivor. ♦ HS (Values significantly higher: >3.0 SD from corresponding baseline) ♦ LS (Values significantly lower: < 3.0 SD from corresponding baseline) ♦ (HS) ♦ Hyperpyrexia (> 3.0°C over baseline) ♦ Hypothermia (>2.0°C under baseline for longer than 2 hrs) ♦ Fever (> 1.5°C over baseline) — BL (Baseline) (Vertical grey dotted line: DPE 0).

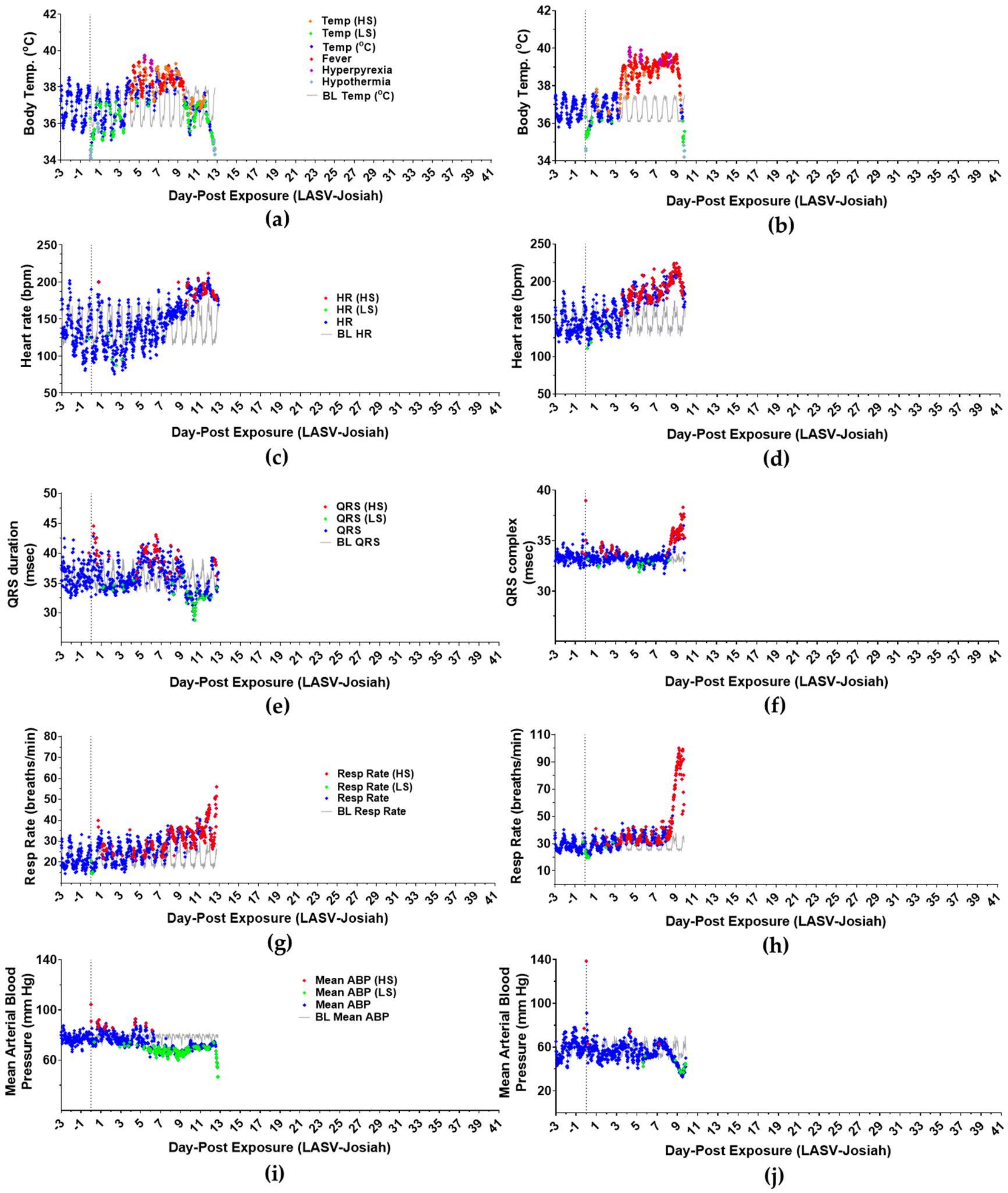

Figure S2. Telemetric assessment of aerosol Lassa virus infected non-survivors. (a) Temperature of D13:non-survivor (b) Temperature of D10:non-survivor.2 (c) Heart rate of D13:non-survivor (d) Heart rate of D10:non-survivor.2 (e) QRS complex of D13:non-survivor (f) QRS complex of D10:non-survivor.2 (g) Respiratory rate of D13:non-survivor (h) Respiratory rate of D10:non-survivor.2 (i) Mean arterial blood pressure of D13:non-survivor (j) Mean arterial blood pressure of D10:non-survivor.2. ♦ HS (Values significantly higher: >3.0 SD from corresponding baseline) ♦ LS (Values significantly lower: < 3.0 SD from corresponding baseline) ♦ (HS) ♦ Hyperpyrexia (> 3.0°C over baseline) ♦ Hypothermia (>2.0°C under baseline for longer than 2 hrs) ♦ Fever (> 1.5°C over baseline) — BL (Baseline) (Vertical grey dotted line: DPE 0).

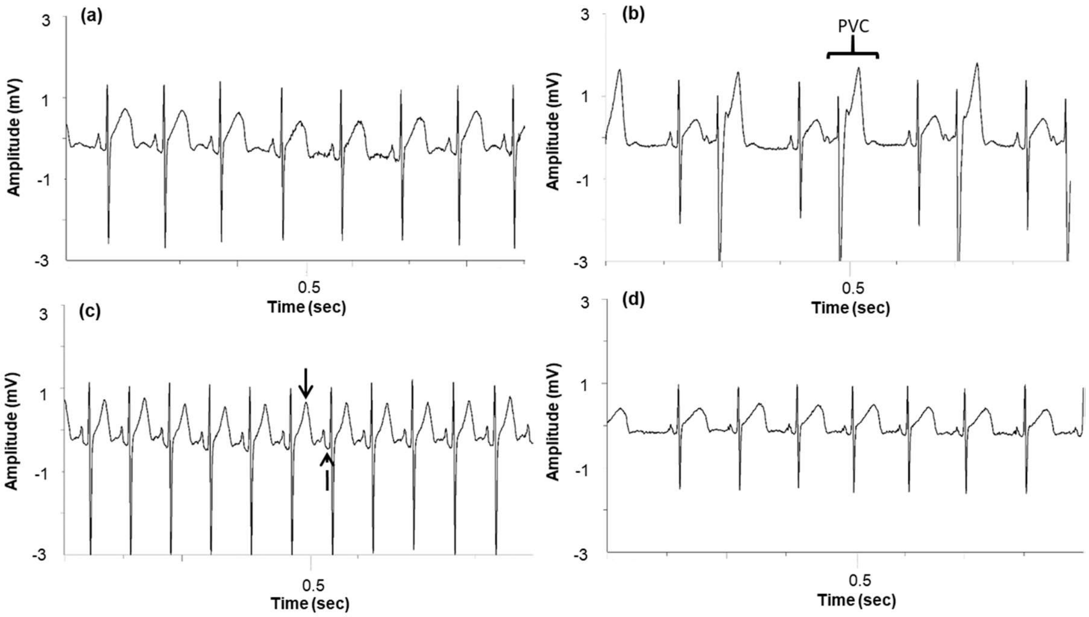

Figure S3. Survivor, ECG highlights. (a) Baseline: prior to challenge. (b) Baseline: prior to challenge with premature ventricular complexes. (bracket). (c) Post-challenge (DPE 8): sinus tachycardia with subtle PR depression (dashed arrow) and peaked T-waves (solid arrow). (d) During recovery from infection (DPE 19): resolution of sinus tachycardia, PR depression, and peaked T-waves. Note decreased voltage of the QRS complex, which could suggest the presence of a pericardial effusion.

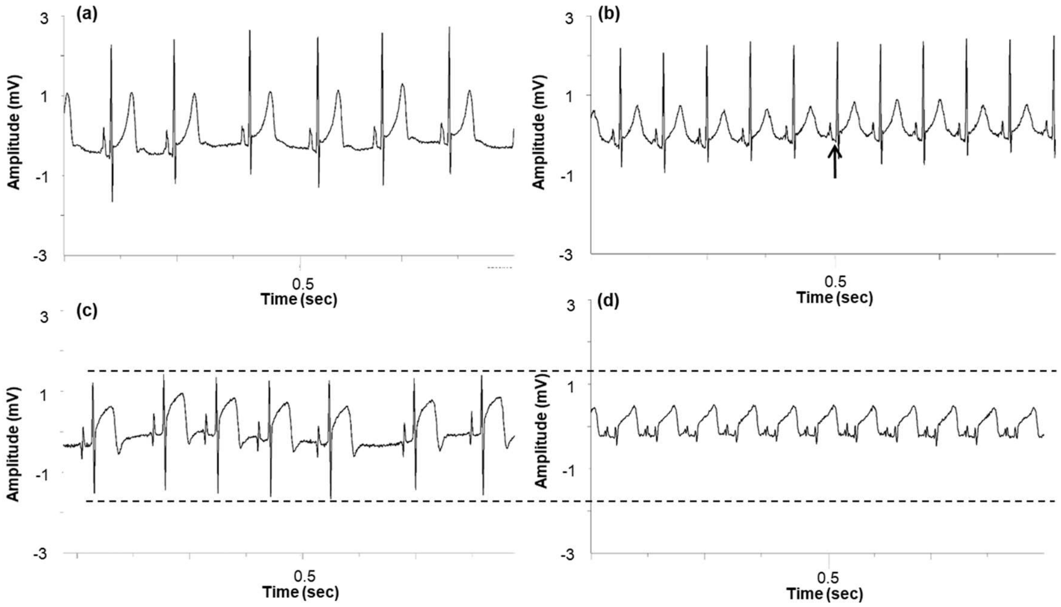

Figure S4. D10:Non-survivor and D10:Non-survivor.2 ECG highlights. (a) ■D10:Non-survivor: prior to challenge. (b) ■D10:Non-survivor post-challenge (DPE 8): sinus tachycardia and subtle PR depression (solid arrow). (c) ▼D10:Non-survivor.2: prior to challenge. (d) ▼D10:Non-survivor (DPE 9): displaying decreased QRS voltage, which could suggest the presence of a pericardial effusion. Dashed line illustrates the QRS voltage difference between DPE 0 and DPE 9.

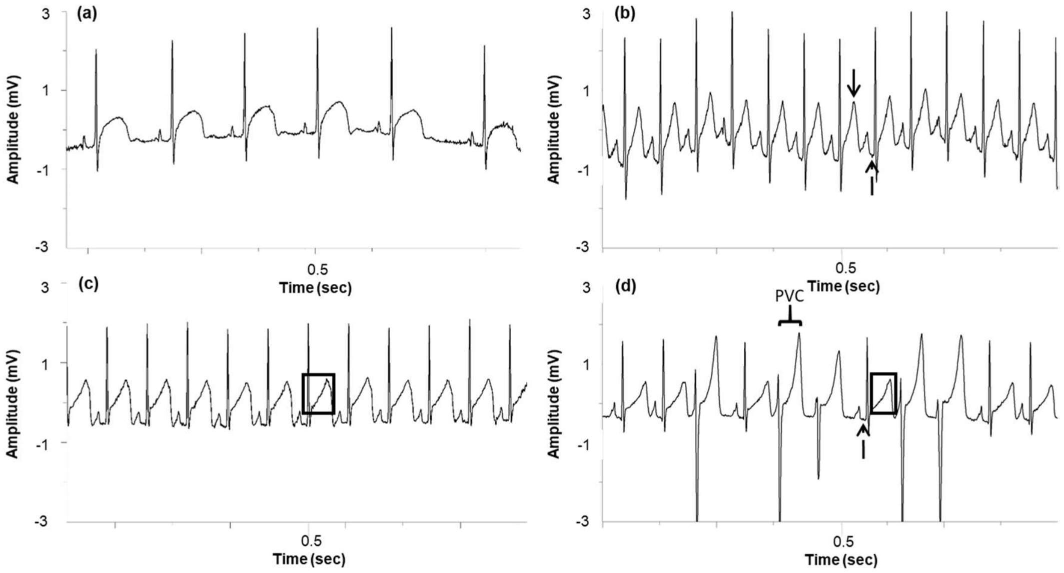

Figure S5. D13:Non-survivor ECG highlights. (a) Baseline: prior to challenge. (b) DPE 6: sinus tachycardia, subtle PR depression (dashed line), and peaked T waves (solid line). (c) DPE 12: sinus tachycardia with up-sloping ST segment (box). (d) DPE 12: similar findings to (b) and(c) with premature ventricular complexes.

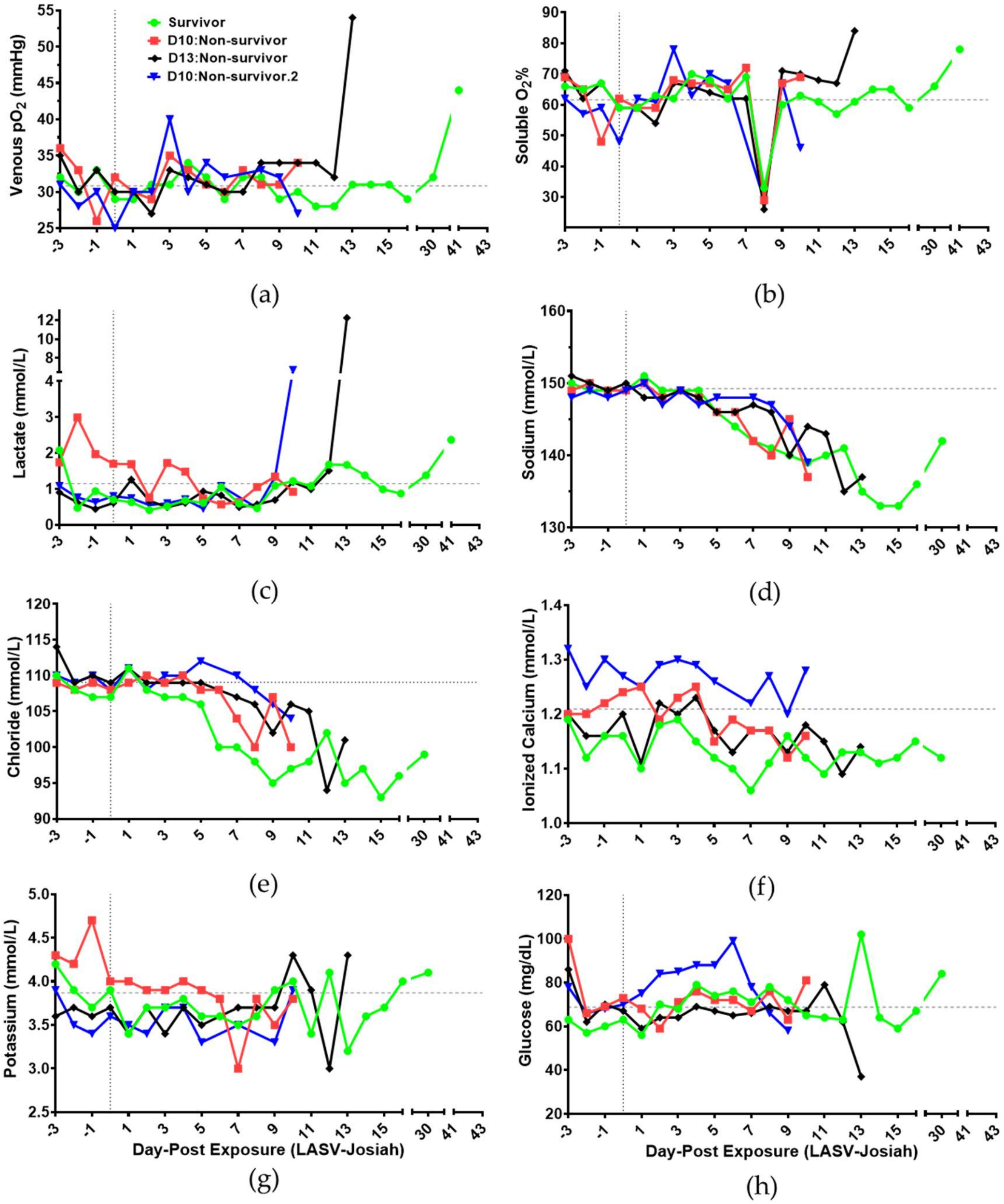

Figure S6. Time course of blood gas and ion-related clinical chemistry in aerosol Lassa virus-challenged cynomolgus macaques. Detection of (a) partial pressure of oxygen: pO_2, (b) soluble O_2%, and (c) lactate in whole blood were conducted using i-Stat 1 CG4+. Plasma (d) sodium, (e) chloride, (f) ionized calcium, (g) potassium, and (h) glucose were detected iStat-1 CHEM8+. (●Survivor) (■D10:Non-survivor) (♦D13:Non-survivor) (▼D10:Non-survivor.2) (Vertical grey dotted line: DPE 0) Grey dashed line is average relative to each animal for DPE -3 to DPE 0.

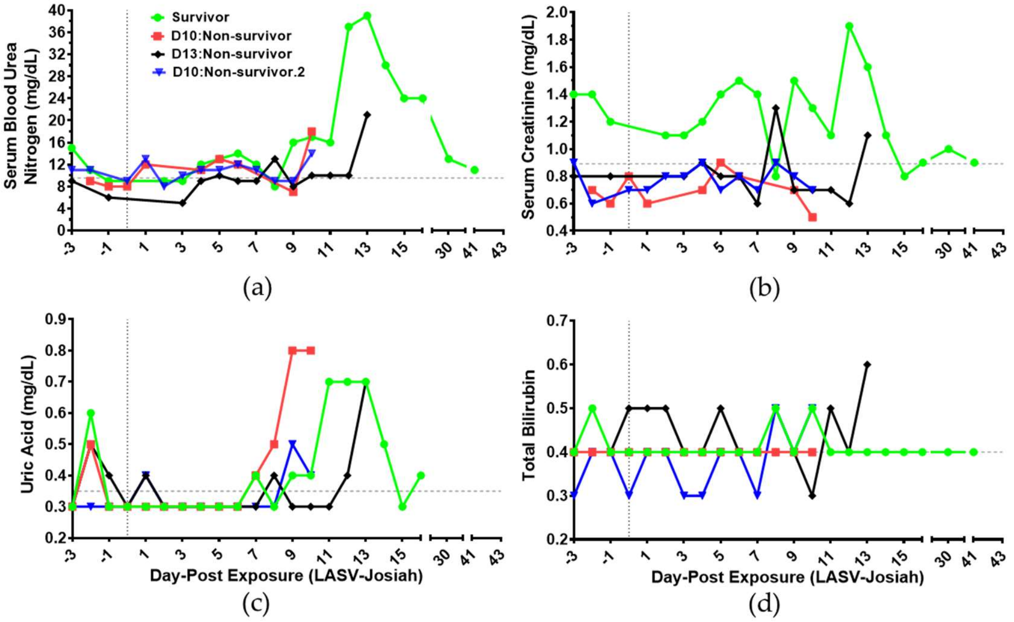

Figure S7. Time course of clinical chemistry in aerosol Lassa virus-challenged cynomolgus macaques. (a) serum blood-urea–nitrogen (BUN), (b) serum creatinine (CRE), (c) plasma uric acid, and (d) plasma total bilirubin (Piccolo). (●Survivor) (■D10:Non-survivor) (♦D13:Non-survivor) (▼D10:Non-survivor.2) (Vertical grey dotted line: DPE 0) Grey dashed line is average relative to each animal for DPE -3 to DPE 0.

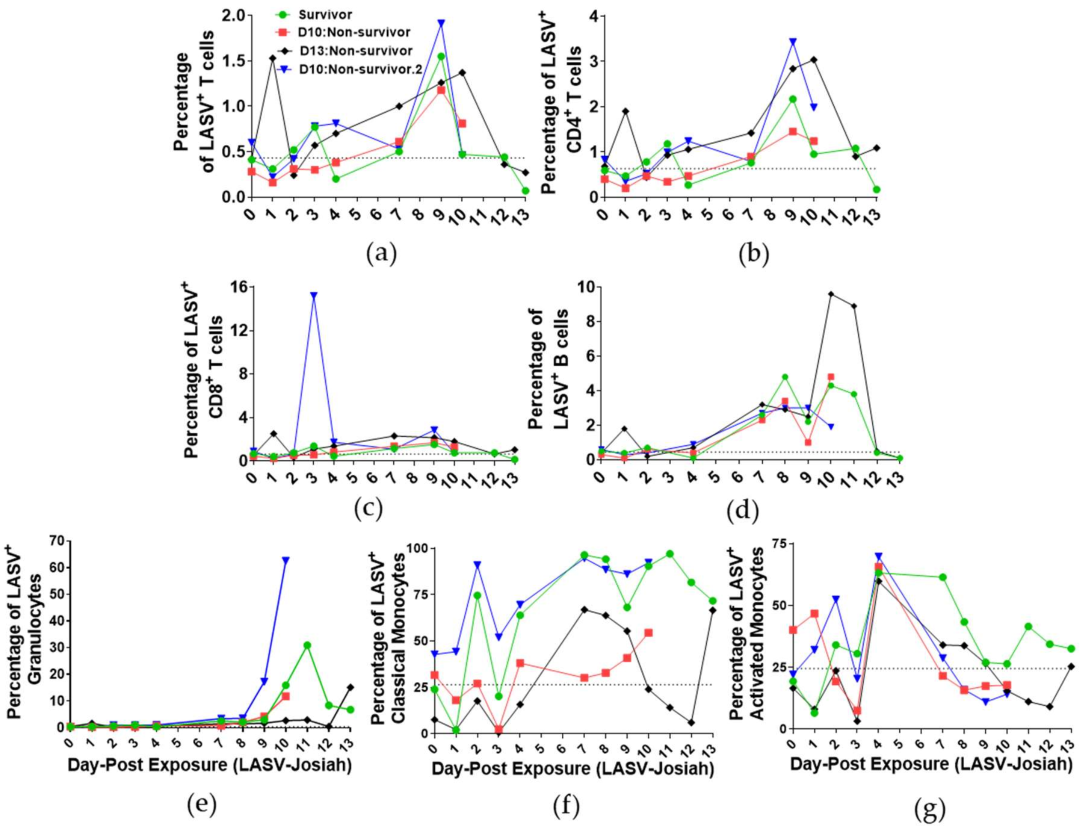

Figure S8. Analysis of LASV-positive lymphocytes in blood of aerosol infected cynomolgus macaques. Percentage of LASV-positive cells, (a) T cells, (b) CD4+ T cells, (c) CD8+ T cells, and (d) B cells, (e) granulocytes, (f) classical monocytes, and (g) activated monocytes detected in blood from day DPE 0 through DPE 13. Horizontal dash line is average at DPE 0. (●Survivor) (■D10:Non-survivor) (♦D13:Non-survivor) (▼ D10:Non-survivor.2)

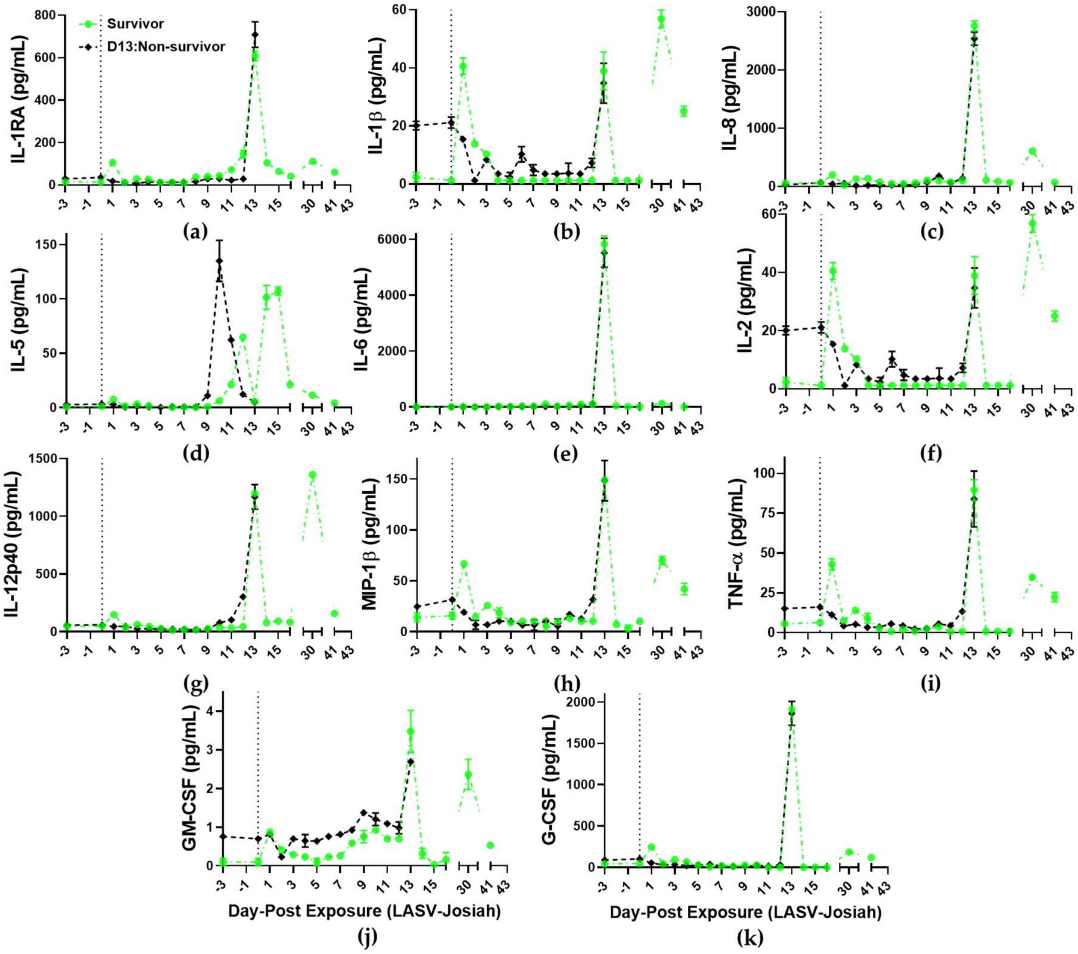

Figure S9. Cytokine analysis of blood from LASV challenged rhesus macaques. Assessment was performed on samples for (a) IL-1RA, (b) IL-1β, (c) IL-2, (d) IL-5, (e) IL-6, (f) IL-8, (g) IL-12p40, (h) MIP-1β, (i) TNFα, (j) GM-CSF, and (k) G-CSF collected 3 days prior to exposure and DPE 0-41. (●Survivor) (♦D13:Non-survivor)

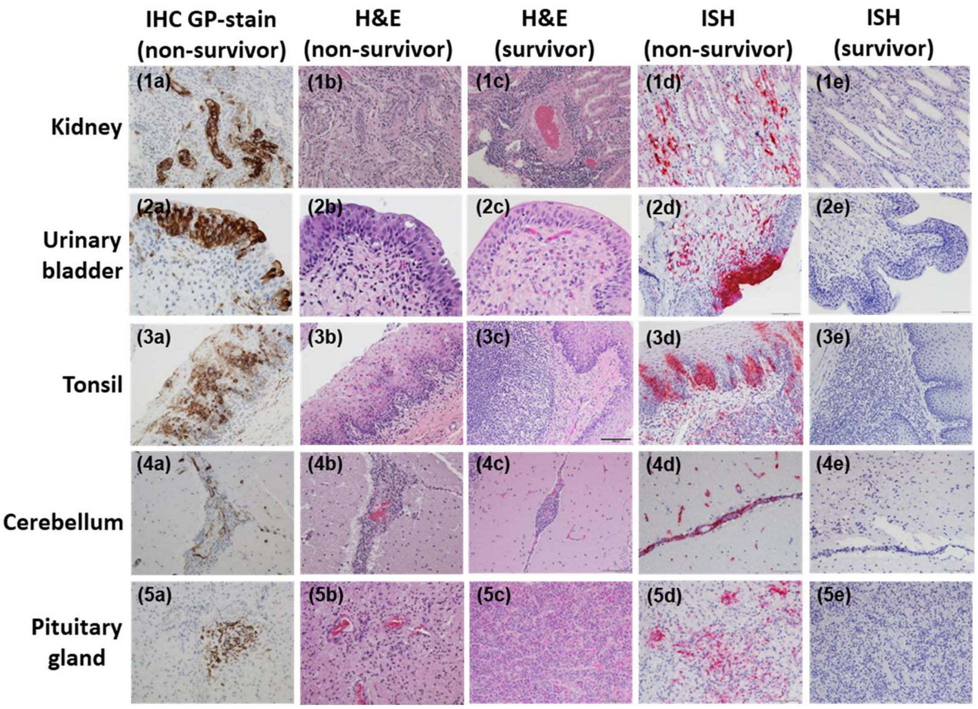

Figure S10. Pathological assessment of tissues of aerosol infected non-human primates. Kidney (1a-1e, 20X), urinary bladder (2a-2e, 20X), tonsil (3a-3e, 20X), cerebellum (4a-4e, 20X), and pituitary gland (5a-5e, 40X). Tissues were from ●survivor (all tissues), ■D10:non-survivor (cerebellum) and ▼ D10:non-survivor (kidney, urinary bladder, tonsil, and pituitary gland). Tissue sections embedded in paraffin were stained for LASV GP2 in non-survivor (IHC-GP: Fig. 1-5a), H&E stain for non-survivor (Fig. 1-5b), and H&E stain for survivor at DPE 41 (Fig. 1-5c). Detection of viral genomic RNA was performed using nucleic acid probe, targeting the polymerase gene with the L segment at sequence 466-1433, in non-survivors at DPE 10(Fig. 1-5d) and absent in the survivor at DPE 41 (Fig. 1-5e) on far right column.
